# Ventriculo-Arterial Coupling: Historical Foundations, Physiological Principles, and Clinical Relevance in Critical Care Hemodynamics

**DOI:** 10.7759/cureus.113201

**Published:** 2026-07-22

**Authors:** Camilo Andres Martínez Buitrago, Nancy Rocio Acosta Murillo

**Affiliations:** 1 Department of Physiology, National University of Colombia, Bogotá, COL

**Keywords:** arterial elastance, critical care hemodynamics, end-systolic elastance, ventriculo-arterial coupling, windkessel model

## Abstract

Ventriculo-arterial coupling describes the interaction between ventricular contractile performance and the arterial system, integrating end-systolic ventricular elastance, arterial elastance, stroke volume generation, and cardiovascular efficiency into a unified physiological framework. Although historically rooted in pressure-volume analysis, time-varying ventricular elastance, and Windkessel-based arterial modeling, the concept has renewed clinical relevance because conventional hemodynamic variables such as blood pressure, cardiac output, and ejection fraction may not adequately reflect the efficiency of ventricular-arterial interaction. This narrative review synthesizes literature published between 1969 and 2026 on the historical foundations, physiological principles, measurement strategies, and clinical applications of ventriculo-arterial coupling, with emphasis on critical care hemodynamics, septic shock, heart failure, perioperative instability, and pediatric cardiovascular assessment. The review highlights that arterial elastance is not simply a measure of arterial wall stiffness but a composite index influenced by vascular resistance, arterial compliance, characteristic impedance, heart rate, systolic timing, and stroke volume. Noninvasive assessment methods have expanded clinical accessibility, yet their interpretation remains limited by methodological assumptions, physiological variability, and incomplete validation in rapidly changing hemodynamic states. Current evidence supports ventriculo-arterial coupling as a valuable interpretive tool for distinguishing impaired contractility, excessive afterload, vasoplegia, preload limitation, and inefficient cardiovascular energy transfer. Standardized measurement protocols, population-specific thresholds, and outcome-based validation are needed before coupling-guided management can be adopted as a therapeutic strategy. The key takeaway is that ventriculo-arterial coupling should currently be used to refine hemodynamic reasoning rather than as an isolated treatment target.

## Introduction and background

Ventriculo-arterial coupling describes the dynamic interaction between ventricular contractile performance and the arterial load against which the ventricle ejects. It is commonly expressed as the ratio of effective arterial elastance (Ea), a composite measure of total arterial load, to end-systolic ventricular elastance (Ees), an index of ventricular contractile properties. The Ea/Ees ratio integrates ventricular mechanics, arterial loading, stroke volume generation, and cardiovascular efficiency within a single physiological framework [1]. The Ea/Ees ratio reflects the balance between arterial load and ventricular contractile performance. Higher values generally indicate relative arterial load excess, reduced ventricular contractility, or both, whereas lower values may reflect reduced arterial load or hyperdynamic ventricular performance. Its interpretation depends on the clinical setting, measurement method, and therapeutic objective. This concept has regained clinical relevance because blood pressure, cardiac output, and systemic vascular resistance do not necessarily demonstrate how efficiently the ventricle transfers mechanical energy to the arterial circulation [1]. Assessment of coupling may help determine whether circulatory dysfunction predominantly reflects impaired contractility, excessive afterload, altered vascular tone, preload limitation, or an inefficient interaction between the ventricle and the arterial system [2].

Physiological variability is essential when interpreting ventriculo-arterial coupling. Ventricular elastance, arterial load, vascular stiffness, and noninvasive estimates of coupling are influenced by age and sex, suggesting that coupling should not be assumed to have a single normal value across all patient populations [3]. This variability is particularly relevant during acute cardiovascular instability, when arterial load and ventricular performance can fluctuate rapidly. In septic shock, myocardial depression, tachycardia, altered vascular impedance, vasoplegia, and vasoactive therapy may collectively produce ventriculo-arterial uncoupling [4]. For example, a patient may reach an apparently acceptable mean arterial pressure after vasopressor administration, yet the associated increase in arterial load may reduce stroke volume or worsen ventricular energetic efficiency when contractile reserve is limited. Thus, conventional hemodynamic targets may appear satisfactory despite inefficient ventricular energy transfer, limited cardiovascular reserve, and persistent tissue hypoperfusion [4].

The physiological basis of ventriculo-arterial coupling emerged from experimental studies of ventricular pressure-volume relationships. Suga demonstrated that the relationship between left ventricular pressure and volume changes continuously throughout the cardiac cycle. Ventricular elastance is therefore time-varying rather than a fixed chamber property, with its maximal value occurring near end-systole [5]. Templeton et al. extended this concept by showing that ventricular stiffness differs during systole and diastole and is influenced by chamber volume and the inotropic state [6]. Collectively, these findings established that ventricular performance cannot be understood through pressure or volume alone but must be interpreted through their interaction with myocardial mechanical properties.

A significant step forward was the work of Sunagawa and colleagues, who integrated ventricular pressure-volume characteristics with arterial loading in an isolated canine ventricular model. Their quantitative framework demonstrated how changes in effective arterial elastance influence stroke volume, end-systolic pressure, and ejection efficiency [7]. This work built upon the characterization by Suga and Sagawa of instantaneous pressure-volume relationships in the supported canine left ventricle, in which end-systolic elastance was proposed as an indicator of ventricular contractile state [8]. Together, these studies established a unified cardiovascular model in which ventricular mechanics and arterial properties are interpreted as components of a coupled system [8].

**Figure 1:**
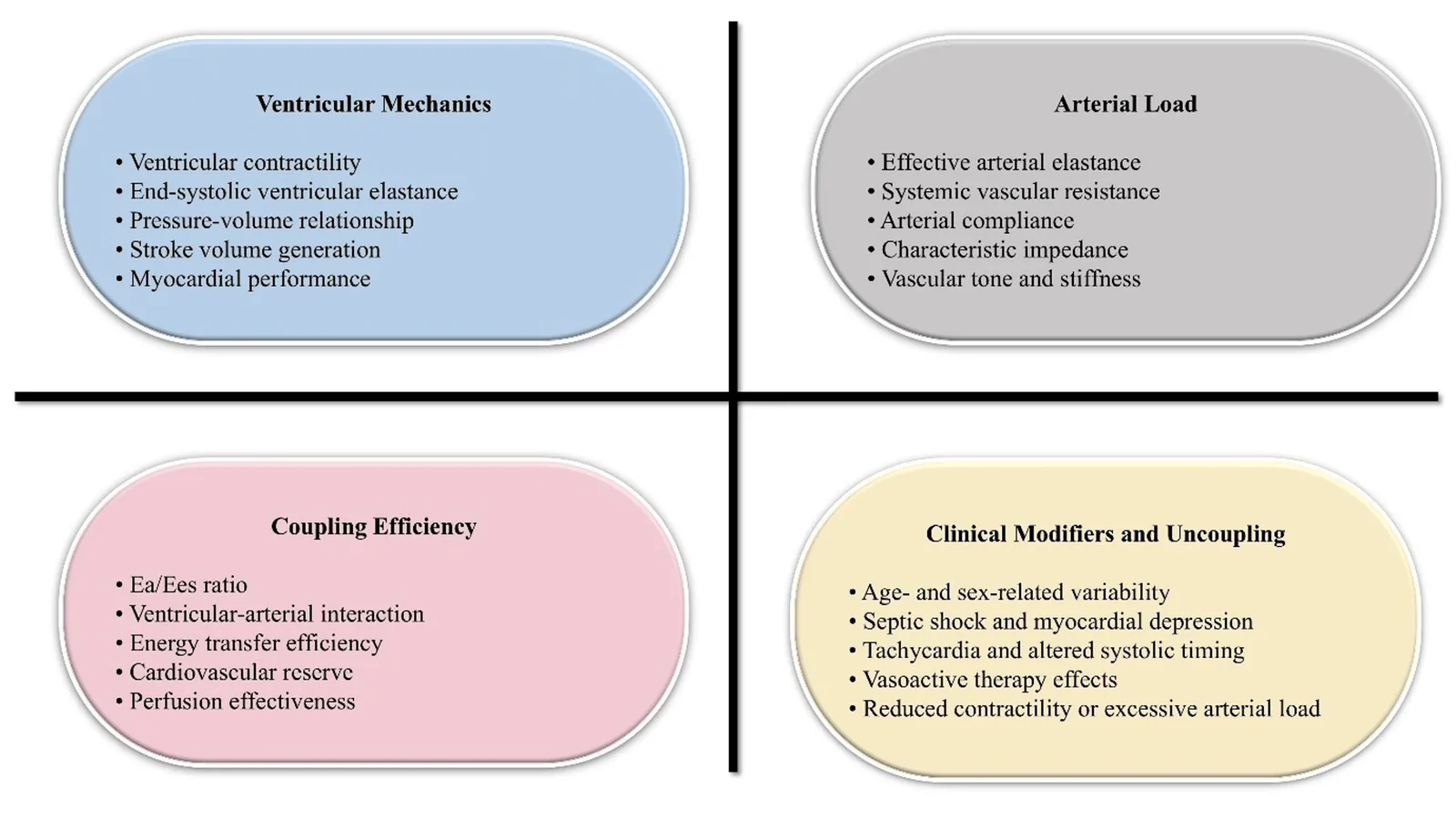
Simplified pressure-volume representation of ventriculo-arterial coupling. The figure shows a simplified left ventricular pressure-volume loop and the relationship between effective arterial elastance (Ea) and end-systolic ventricular elastance (Ees). Ees is represented by the slope of the end-systolic pressure-volume relationship and reflects ventricular contractile properties. Ea represents the net arterial load opposing ventricular ejection and may be approximated from end-systolic pressure and stroke volume. The Ea/Ees ratio describes the balance between ventricular contractile performance and arterial loading. Higher ratios generally indicate relative arterial load excess, reduced ventricular contractility, or both, whereas lower ratios may reflect reduced arterial load or hyperdynamic ventricular function.

A clear distinction among elastance, elasticity, stiffness, and compliance is necessary for accurate interpretation. Elastance describes the change in pressure associated with a change in volume and is the inverse of compliance, whereas stiffness and elasticity refer to related but distinct material and structural properties. The mechanical properties of blood vessels are influenced by wall composition, geometry, loading, and viscoelastic properties, and blood vessels behave as nonlinear biological structures [9]. Tissue stiffness is also context-dependent and reflects the interaction of cellular behavior, mechanical loading, and extracellular matrix organization rather than a single fixed physical property [10]. These distinctions are important because effective arterial elastance does not directly measure arterial wall stiffness. Instead, it is a lumped, or composite, index influenced by systemic vascular resistance, arterial compliance, characteristic impedance, heart rate, systolic ejection timing, and stroke volume [11]. The main determinants and clinical modifiers of ventriculo-arterial coupling are summarized in Figure 1.

Clinical application has subsequently expanded through noninvasive approaches based on echocardiography, arterial pressure, stroke volume, and derived hemodynamic indices [12]. These methods have improved the feasibility of coupling assessment in cardiology, perioperative medicine, and critical care, although each depends on assumptions regarding ventricular pressure, volume, timing, and loading conditions [12]. The arterial component is associated with the Windkessel function of central arteries, which can be understood as the capacity of large elastic arteries to temporarily store part of the energy generated during systole and release it during diastole. This buffering function reduces excessive pulsatility and helps maintain forward blood flow between ventricular ejections [13]. The concept dates back to Otto Frank's analysis of the arterial pulse, which is fundamental to understanding arterial compliance, resistance, and pressure-flow relationships [14]. Modern models improve the representation of the aorta by incorporating resistance, compliance, impedance, and pulsatile flow [15].

Important knowledge gaps remain despite these physiological and methodological advances. Universally accepted thresholds for normal and abnormal ventriculo-arterial coupling have not been established across age groups, sexes, disease phenotypes, and measurement techniques. Noninvasive methods require further validation against invasive pressure-volume analysis, particularly during rapidly changing hemodynamic states. Evidence also remains insufficient to determine whether coupling-guided therapy improves clinically meaningful outcomes during septic shock, pediatric critical illness, perioperative instability, or vasoactive-drug titration [16]. This narrative review aims to integrate the historical foundations, physiological principles, measurement strategies, clinical applications, methodological limitations, and future research priorities of ventriculo-arterial coupling as a framework for interpreting cardiovascular performance.

Methodology

Review Design

This manuscript was developed as a narrative review of ventriculo-arterial coupling, with an emphasis on its historical development, physiological basis, measurement strategies, and clinical relevance in cardiovascular and critical care medicine. A narrative approach was selected because the topic spans foundational experimental physiology, mathematical modeling, translational cardiovascular assessment, and emerging bedside applications. The aim was to provide an integrated conceptual and clinical synthesis rather than to answer a narrowly defined intervention question. PRISMA methodology and its components, including protocol registration, systematic database screening, eligibility flow diagrams, and systematic-review reporting procedures, were not used because this manuscript was not designed as a systematic review. The conduct and reporting of the review were informed by principles relevant to high-quality narrative reviews, including a clearly stated objective, transparent description of the literature search, appropriate referencing, balanced scientific reasoning, and presentation of clinically relevant evidence. A formal SANRA score was not assigned.

Literature Search Strategy

Relevant literature published between 1969 and 2026 was identified through targeted searches of PubMed, Google Scholar, Scopus, and Web of Science, together with reference-list screening. The final literature search was completed on June 10, 2026. This time frame was selected to capture the historical development of ventricular elastance and pressure-volume analysis, the evolution of arterial elastance and Windkessel-based models, and contemporary clinical applications of ventriculo-arterial coupling in critical care, septic shock, heart failure, perioperative hemodynamics, and pediatric cardiovascular assessment. Search terms included “ventriculo-arterial coupling,” “ventricular-arterial coupling,” “arterial elastance,” “end-systolic elastance,” “pressure-volume relationship,” “Windkessel model,” “dynamic arterial elastance,” “septic shock,” “heart failure,” “critical care hemodynamics,” “non-invasive assessment,” and “pediatric cardiovascular physiology.” The terms were used individually and in combinations with Boolean operators such as “AND” and “OR” to connect the principal physiological concept with specific measurement methods, populations, and clinical settings. Reference lists of relevant original studies, reviews, and consensus documents were also examined to identify foundational or clinically important publications not retrieved during the initial searches. The search was purposive rather than exhaustive, consistent with the narrative design of the review.

Eligibility Criteria

Eligible publications included foundational experimental studies, human physiological investigations, observational studies, interventional studies, methodological and validation studies, consensus documents, and relevant reviews that contributed directly to the historical, physiological, measurement-related, or clinical interpretation of ventriculo-arterial coupling. Adult and pediatric literature was considered when it addressed ventricular elastance, effective arterial elastance, pressure-volume analysis, Windkessel physiology, dynamic arterial elastance, ventricular-vascular interaction, or clinical applications in heart failure, septic shock, perioperative medicine, and critical care. Articles were excluded when they were unrelated to ventricular-arterial interaction, lacked sufficient physiological or methodological relevance to the review objective, duplicated evidence already represented by a more complete source, or consisted primarily of unsupported opinion without substantive mechanistic or clinical information.

Article Selection and Evidence Synthesis

Articles were prioritized according to conceptual relevance, methodological clarity, clinical applicability, and contribution to the interpretation of ventriculo-arterial coupling. Experimental studies were used to explain mechanistic principles, while clinical studies, reviews, and consensus literature were used to contextualize bedside interpretation and therapeutic relevance. Potentially relevant publications were assessed initially by title and abstract, followed by full-text evaluation when the article appeared to contribute directly to one or more predefined themes of the review. Both authors participated in the interpretation and selection of the literature, and disagreements regarding inclusion, relevance, or interpretation were resolved through discussion and consensus. Selection was purposive and theme-based rather than based on exhaustive systematic screening. The evidence was synthesized qualitatively and thematically rather than statistically. Greater interpretive weight was given to studies that directly assessed ventricular or arterial mechanics, used validated measurement methods, included clearly defined populations, and reported findings consistent with related physiological or clinical evidence. Differences among studies were considered according to study design, population, ventricular or vascular territory, measurement technique, disease state, and therapeutic context. Inconsistent findings were presented as areas of uncertainty rather than combined into a single numerical estimate. No meta-analysis, pooled estimates, or quantitative synthesis was performed because of substantial heterogeneity in study design, population, measurement technique, and clinical setting. No p-values, confidence intervals, pooled effect estimates, meta-regression, or formal statistical comparisons were generated for this review. Evidence was synthesized thematically into sections addressing historical development, physiological principles, measurement approaches, septic shock, heart failure, dynamic arterial elastance, pediatric validation, limitations, and future directions.

Quality and Risk-of-Bias Appraisal

The included literature was appraised by the authors for methodological limitations, relevance to the review objective, and potential risk of bias. The appraisal considered study design, population characteristics, sample size, validity of the measurement method, use of invasive or noninvasive assessment, control of relevant confounding factors, consistency with related evidence, clinical applicability, and limitations acknowledged by the original investigators. Experimental and physiological studies were also assessed according to the appropriateness of the model and the extent to which their findings could be translated to human or critically ill populations. Given the narrative design and heterogeneity of included sources, no single standardized risk-of-bias instrument was applied across all article types. A uniform instrument was considered inappropriate because the evidence base included animal experiments, invasive physiological investigations, validation studies, observational cohorts, interventional studies, reviews, and consensus documents requiring different appraisal frameworks. Any differences in interpretation or inclusion were resolved through discussion and consensus between the authors.

## Review

Septic shock, vasoactive therapy, and ventriculo-arterial decoupling

Septic shock is one of the most clinically relevant settings in which ventriculo-arterial coupling may become markedly impaired [16]. Human studies have demonstrated ventriculo-arterial uncoupling during septic shock, reflecting an imbalance between ventricular contractile performance and arterial load [17]. This condition cannot be understood solely as a disorder of vascular tone because myocardial depression, vasoplegia, tachycardia, altered vascular impedance, and vasoactive therapy may interact simultaneously. Mean arterial pressure may therefore appear adequate despite inefficient ventricular energy transfer, reduced stroke volume reserve, or persistent tissue hypoperfusion.

Heart-rate modulation may influence this interaction in selected patients. In septic shock, heart-rate reduction with esmolol has been associated with improved arterial elastance [18]. This finding suggests that persistent tachycardia may contribute to mechanical inefficiency in some patients rather than functioning exclusively as a compensatory response. The available evidence is observational, and beta-blockade should not be generalized to unstable patients without careful assessment of ventricular function, perfusion, and compensatory reserve.

Vasoactive and emerging hemodynamic therapies may also modify coupling through their effects on vascular tone, myocardial contractility, arterial compliance, and total arterial load [19]. Their effects should therefore be interpreted according to changes in forward flow and cardiovascular efficiency rather than arterial pressure alone. Noninvasive approaches, including aortic wave-intensity analysis combined with central blood pressure and phase-contrast cardiovascular magnetic resonance, may provide additional characterization of ventriculo-arterial interaction without invasive pressure-volume measurements [20]. These techniques remain technically demanding and require broader validation before routine use in rapidly changing septic shock states.

Norepinephrine response and early resuscitation

Ventriculo-arterial coupling may help explain why vasopressor-induced increases in arterial pressure do not always improve forward flow [21]. In septic shock, left ventriculo-arterial coupling has been evaluated as a predictor of stroke-volume response to norepinephrine [22]. When ventricular contractile reserve is limited, an increase in aortic pressure may occur without producing a corresponding improvement in stroke volume. A pressure response should therefore not be interpreted automatically as evidence of improved cardiovascular efficiency.

**Figure 2:**
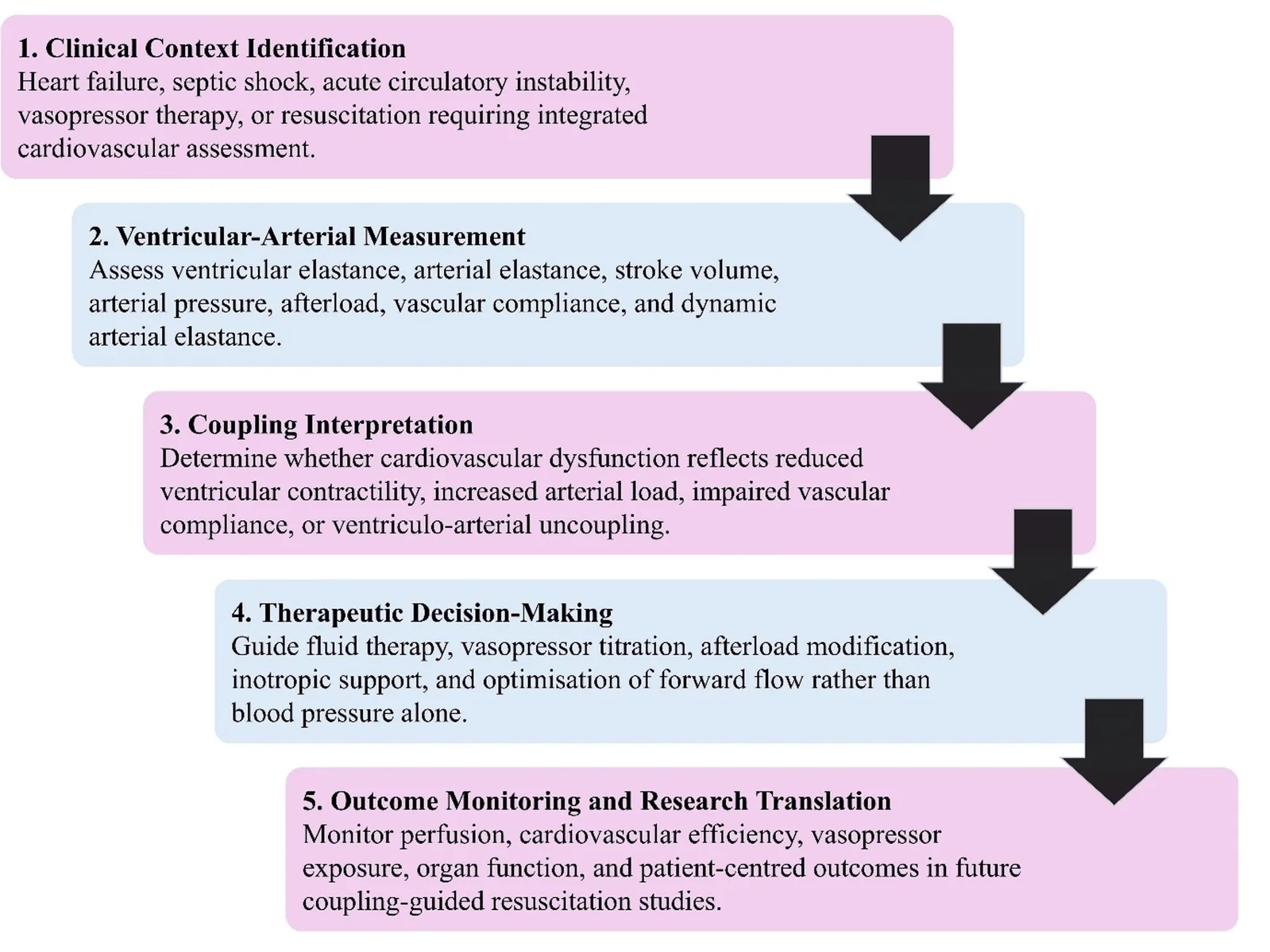
Clinical application pathway for ventriculo-arterial coupling assessment.

The related role of dynamic arterial elastance in distinguishing volume responsiveness from arterial pressure responsiveness is discussed separately in the dedicated section below [23]. Early studies have also examined ventriculo-arterial coupling as a potential resuscitation target in septic shock [24]. These findings support physiological plausibility but do not yet establish coupling-guided management as a standard therapeutic strategy. Larger trials are required to determine whether this approach improves organ perfusion, reduces vasopressor exposure, or produces better patient-centered outcomes. The stepwise clinical integration of ventriculo-arterial coupling in acute resuscitation is summarized in Figure 2.

Noninvasive assessment and physiological variability

Ventriculo-arterial coupling is difficult to assess during rapidly changing hemodynamic states because preload, afterload, heart rate, vascular tone, and contractility may change within minutes. A single measurement may therefore represent only a transient cardiovascular condition rather than a stable physiological relationship [25]. This limitation is particularly relevant during shock, perioperative instability, and vasoactive-drug titration, when an increase in arterial pressure or stroke volume may occur without a corresponding improvement in mechanical efficiency [26]. Coupling should therefore be interpreted serially, before and after a defined intervention, rather than from an isolated measurement.

The principal challenge in translating ventriculo-arterial coupling from an experimental construct to clinical practice is obtaining reliable noninvasive measurements [27]. Invasive pressure-volume analysis remains the reference method but is not suitable for repeated bedside assessment [28]. Single-beat methods can estimate left ventricular end-systolic elastance without conductance catheterization and may improve the feasibility of coupling assessment in cardiology, perioperative medicine, and critical care [29]. Serial assessment is particularly important during acute circulatory failure, when therapeutic interventions and rapidly changing loading conditions may alter coupling over short periods. These methods remain dependent on assumptions regarding ventricular pressure, volume, timing, and loading conditions, all of which may be unstable during acute circulatory failure.

Universal thresholds are further limited by physiological variability. Normal human coupling relationships demonstrate that ventriculo-arterial interaction is context dependent rather than fixed [30]. Age-related arterial stiffening, altered ventricular compliance, and reduced contractile reserve may shift coupling even in the absence of overt systolic dysfunction [31]. Coupling also changes with physiological demand because exercise requires coordinated adaptation of heart rate, ventricular contractility, vascular tone, and pulsatile arterial load [32]. Noninvasive coupling assessment is therefore best used as a serial and context-dependent interpretive tool rather than as an isolated diagnostic marker. Table 1 summarizes the principal clinical applications and limitations of noninvasive assessment.

**Table 1:** Key considerations in noninvasive assessment of ventriculo-arterial coupling.

Assessment domain	Clinical relevance	Main interpretive limitation	References
Single-beat estimation of end-systolic elastance	Enables approximation of ventricular contractile properties without invasive pressure-volume catheterization	Depends on assumptions regarding ventricular pressure, volume, timing, and loading conditions	[29]
Normal human coupling relations	Demonstrates that coupling reflects interaction between ventricular performance and arterial properties	Limits the use of a single universal normal range	[30]
Age-related coupling changes	Explains how arterial stiffening and altered ventricular compliance affect cardiovascular reserve	Age-related shifts may mimic or mask disease-related coupling abnormalities	[31]
Coupling during physiological demand	Shows that ventricular-arterial interaction changes during exercise and stress	Resting estimates may underestimate abnormalities that appear only under increased demand.	[32]

Ventriculo-arterial coupling in heart failure

Ventriculo-arterial coupling provides a useful framework for understanding heart failure because cardiovascular performance depends on both ventricular contractile reserve and the arterial load against which the ventricle ejects. Impaired myocardial function, increased arterial stiffness, reduced vascular compliance, and abnormal wave reflections may act together to decrease mechanical and energetic efficiency [33]. Ejection fraction alone may not adequately represent the ability of the ventricle to adapt to arterial load. Patients with similar ejection fractions may therefore have different hemodynamic profiles, exercise capacities, cardiovascular reserve, and risks of decompensation.

Abnormal coupling in chronic heart failure has been associated with ventricular remodeling and prognosis, indicating that ventricular-arterial interaction reflects more than isolated systolic function [34]. These associations support the value of coupling for disease characterization and risk assessment. Evidence that coupling-directed interventions improve survival, hospitalization, or functional outcomes remains limited.

This distinction is particularly relevant in heart failure with preserved ejection fraction. Combined ventricular systolic stiffening and arterial stiffening may impair systolic and diastolic reserve without reducing the ejection fraction [35]. This interaction provides a physiological explanation for blood pressure sensitivity, exertional intolerance, and limited hemodynamic adaptability. Chronic heart failure should therefore be viewed as a continuum of ventricular-vascular abnormalities rather than as a single pattern of coupling failure [36]. The principal current value of ventriculo-arterial coupling lies in clarifying disease mechanisms and phenotypes. Routine clinical implementation requires standardized measurement methods, phenotype-specific reference values, and evidence that coupling-guided management improves clinically meaningful outcomes.

Windkessel physiology, pressure-volume analysis, and clinical translation

The Windkessel model provides a simplified representation of the arterial contribution to ventriculo-arterial coupling. The arterial system is not a rigid conduit. Large elastic arteries temporarily store part of the energy generated during systole and release it during diastole, thereby reducing pulsatile flow and maintaining forward perfusion between ventricular ejections [37]. Effective arterial elastance reflects more than systemic vascular resistance and is influenced by arterial compliance, characteristic impedance, heart rate, stroke volume, and ventricular ejection timing. Simplified Windkessel models may not fully represent regional vascular heterogeneity, wave reflections, or disease-related structural changes in the arterial system.

Invasive pressure-volume analysis remains the most direct method for evaluating ventricular mechanics and ventriculo-arterial interaction. It permits detailed assessment of ventricular elastance, loading dependence, pressure-volume relationships, and energetic efficiency [38]. Its clinical feasibility is limited by the need for invasive catheterization, making it unsuitable for routine or repeated monitoring in most critically ill patients.

Clinical translation, therefore, depends on pragmatic monitoring approaches in perioperative and intensive care settings. Ventriculo-arterial coupling may assist in interpreting hemodynamic instability during fluid shifts, vasopressor or inotrope administration, anesthesia, and mechanical ventilation [39]. Its principal value lies in determining whether an intervention improves forward flow and cardiovascular efficiency or merely raises arterial pressure. Noninvasive assessment of ventricular elastance is feasible in the intensive care unit, but agreement among available methods remains inconsistent [40]. Standardized acquisition protocols, serial interpretation, and outcome-based validation are required before coupling can be used as an independent therapeutic target in critical care.

Dynamic arterial elastance, fluid responsiveness, and sepsis management

Dynamic arterial elastance provides a functional estimate of whether an increase in stroke volume is likely to produce a clinically meaningful increase in arterial pressure. It is commonly calculated as the ratio of pulse pressure variation to stroke volume variation and has been proposed as a predictor of arterial pressure response to volume loading in preload-dependent patients [41]. Fluid responsiveness and pressure responsiveness are not equivalent. An increase in stroke volume after fluid administration may fail to restore arterial pressure when vascular tone, arterial loading, or ventriculo-arterial energy transfer remains impaired.

Dynamic arterial elastance has also been evaluated in spontaneously breathing patients, in whom prediction is more difficult because respiratory changes in preload are less controlled than during mechanical ventilation [42]. It may help relate volume responsiveness to vascular pressure responsiveness and distinguish patients who may benefit from fluid administration from those who may require adjustment of vasopressor therapy. Reliability may be reduced by arrhythmias, spontaneous respiratory effort, variations in tidal volume, right ventricular dysfunction, vasoplegia, and differences among monitoring platforms. Dynamic arterial elastance should therefore be interpreted as a context-dependent hemodynamic signal rather than as a universal decision rule.

In septic shock, hemodynamic management requires integration of perfusion targets, fluid therapy, vasopressor use, cardiac function, and repeated reassessment [43]. Dynamic arterial elastance and ventriculo-arterial coupling may help determine whether hypotension predominantly reflects preload insufficiency, vascular hyporesponsiveness, impaired myocardial contractility, or inefficient ventriculo-arterial interaction. These indices remain adjuncts to comprehensive bedside assessment and should not be used as stand-alone therapeutic endpoints. Their broader clinical adoption requires standardized measurement conditions and prospective evidence demonstrating improvement in organ perfusion, treatment exposure, or patient-centered outcomes. Table 2 summarizes the clinical applications and principal limitations of dynamic arterial elastance in fluid administration, arterial pressure assessment, sepsis management, and pediatric ventriculo-arterial assessment.

**Table 2:** Dynamic arterial elastance, fluid responsiveness, and sepsis management.

Concept	Clinical relevance	Main interpretive limitation	References
Dynamic arterial elastance	Estimates whether an increase in stroke volume is likely to produce a meaningful rise in arterial pressure after volume loading.	Fluid responsiveness does not necessarily indicate pressure responsiveness when vascular tone or arterial load is impaired.	[41]
Assessment of spontaneously breathing patients	Extends pressure-response assessment beyond controlled mechanical ventilation.	Respiratory variability, spontaneous effort, arrhythmias, and tidal volume changes may reduce reliability.	[42]
Sepsis and septic shock resuscitation	Places dynamic arterial elastance within broader resuscitation involving fluids, vasopressors, perfusion targets, and repeated reassessment.	Should not replace integrated assessment of perfusion, cardiac function, and therapeutic response.	[43]

Pediatric validation and future clinical integration

Pediatric application of ventriculo-arterial coupling requires dedicated evaluation because cardiovascular mechanics differ substantially between children and adults. Developmental variations in heart rate, ventricular size, myocardial compliance, vascular resistance, arterial elasticity, and cardiovascular reserve influence ventriculo-arterial interaction [1,2]. Population-specific interpretation is further supported by age- and sex-related variability in noninvasive coupling estimates [3]. In pediatric critical care, interpretation is particularly complex because ventricular performance and arterial load may change rapidly during shock, postoperative instability, mechanical ventilation, vasoactive-drug administration, and fluctuating loading conditions [11,25].

Noninvasive assessment is especially important in children because invasive pressure-volume analysis, despite remaining the physiological reference standard, is impractical for routine or repeated bedside use [27,38]. Echocardiographic techniques have increased the feasibility of estimating ventricular mechanics and arterial loading, although these methods depend on assumptions regarding ventricular pressure, volume, timing, and loading conditions [29,40]. In pediatric pulmonary arterial hypertension, impaired ventricular-vascular coupling has been associated with adverse clinical outcomes, supporting its potential prognostic relevance in selected populations [44]. This evidence primarily concerns right ventricular-pulmonary arterial coupling and should not be interpreted as directly equivalent to systemic left ventriculo-arterial coupling. Simultaneous echocardiographic and conductance-catheter assessment has also demonstrated the feasibility of evaluating left ventricular mechanics in children, although available validation studies remain small and encompass limited disease spectra and severity ranges [45]. Interpretation may be further affected by small ventricular volumes, high heart rates, limited acoustic windows, sedation, mechanical ventilation, and vasoactive therapy.

Future clinical implementation requires age-specific reference ranges, disease-specific thresholds, standardized echocardiographic acquisition protocols, and validation in pediatric critical care, congenital heart disease, pulmonary hypertension, and perioperative populations. Coupling indices should be interpreted alongside ventricular function, preload responsiveness, arterial pressure response, vascular tone, and the patient's therapeutic trajectory [41,42]. Pediatric ventriculo-arterial coupling remains a physiologically informative but clinically incompletely validated framework. It may currently support mechanistic interpretation and risk stratification in selected settings, but coupling-guided interventions cannot be recommended for routine pediatric practice until prospective studies demonstrate reproducibility, therapeutic utility, and improvement in clinically meaningful outcomes.

Limitations and future directions

This narrative review has a few limitations. It did not use formal systematic screening, standardized risk-of-bias assessment, or quantitative synthesis, which limits reproducibility and may introduce selection bias. The evidence base is heterogeneous and includes animal experiments, invasive pressure-volume studies, noninvasive validation studies, consensus statements, and early clinical investigations. These sources provide complementary physiological insights but differ substantially in design, population, measurement technique, and clinical applicability.

Ventriculo-arterial coupling is also assessed using multiple approaches, including invasive pressure-volume analysis, echocardiographic estimation, effective arterial elastance, dynamic arterial elastance, and cardiovascular magnetic resonance. This methodological diversity limits direct comparison across studies and complicates the definition of clinically meaningful thresholds. Findings derived from stable experimental conditions may not be fully generalizable to critically ill patients, in whom preload, afterload, vascular tone, heart rate, and contractility can change rapidly.

Future research should prioritize standardized acquisition and calculation protocols and establish clinically relevant thresholds across age groups, sexes, disease phenotypes, and care settings. Larger prospective studies are required to compare noninvasive methods with invasive pressure-volume analysis in septic shock, heart failure, perioperative instability, and pediatric critical care. Outcome-based trials should determine whether coupling-guided management improves organ perfusion, reduces vasopressor exposure, enhances cardiovascular efficiency, shortens intensive care unit stay, or reduces mortality.

Bedside echocardiography, continuous hemodynamic monitoring, and computational models may support real-time interpretation, but these approaches require further validation before routine implementation. The central challenge is to translate ventriculo-arterial coupling from a physiologically coherent concept into a reproducible, clinically practical, and outcome-relevant decision-support framework.

## Conclusions

Ventriculo-arterial coupling integrates ventricular contractile performance with arterial load and stroke volume generation, providing a physiological framework that extends beyond blood pressure, cardiac output, and ejection fraction alone. It may help distinguish impaired contractility, excessive afterload, vasoplegia, preload limitation, and inefficient ventricular-arterial interaction in septic shock, heart failure, perioperative instability, and selected pediatric conditions. Its principal current value is as an adjunctive tool for interpreting hemodynamic status and treatment response rather than as an independent therapeutic target. Clinical application remains limited by methodological variability, simplified measurement assumptions, and incomplete validation of noninvasive indices in rapidly changing states. Future research should standardize measurement protocols, establish population-specific thresholds, and determine whether coupling-guided management improves perfusion, therapeutic response, and patient-centered outcomes.
